# Influence of *Azadirachta indica* and *Cnidoscolus angustidens* aqueous extract on cattle ruminal gas production and degradability *in vitro*

**DOI:** 10.3389/fvets.2023.1090729

**Published:** 2023-05-17

**Authors:** Mona M. M. Y. Elghandour, Néstor Acosta-Lozano, Tonantzin Díaz Alvarado, Ezequias Castillo-Lopez, Moises Cipriano-Salazar, Marcos Barros-Rodríguez, Udoh Akpan Inyang, Rayudika Aprilia Patindra Purba, Abdelfattah Z. M. Salem

**Affiliations:** ^1^Facultad de Medicina Veterinaria y Zootecnia, Universidad Autónoma del Estado de México, Toluca, Mexico; ^2^Centro de Investigaciones Agropecuarias, Facultad de Ciencias Agrarias, Universidad Estatal Península de Santa Elena, Santa Elena, Ecuador; ^3^Facultad de Estudios Superiores Cuautitlan, Universidad Nacional Autonoma de Mexico (UNAM), Cuautitlan, Mexico; ^4^Department of Farm Animals and Public Health, Institute of Animal Nutrition and Functional Plant Compounds, University of Veterinary Medicine Vienna, Vienna, Austria; ^5^Facultad de Medicina Veterinaria y Zootecnia No. 1, Universidad Autónoma de Guerrero, Guerrero, Mexico; ^6^Facultad de Ciencias Agropecuarias, Universidad Técnica de Ambato, Sector el Tambo-La Universidad, vía a Quero, Cevallos, Ambato, Ecuador; ^7^Department of Animal Science, Faculty of Agriculture, University of Uyo, Uyo, Nigeria; ^8^School of Animal Technology and Innovation, Institute of Agricultural Technology, Suranaree University of Technology, Nakhon Ratchasima, Thailand

**Keywords:** *in vitro* gas production, greenhouse gases, plant polyphenols, fermentative characteristics, cattle

## Abstract

**Introduction:**

Mitigation of ruminant greenhouse gas (GHG) emissions is crucial for more appropriate livestock production. Thus, there is a need of further research evaluating feed supplementation strategies to mitigate enteric GHG emissions and other gases produced within the rumen.

**Methods:**

This study was conducted as a completely randomized experimental design to determine the effectiveness of liquid extracts from *A. indica* (AZI), *C. angustidens* (CNA), or their combination (Mix. 1:1) at dosages of 0, 36, 72, and 108 mg of liquid extract/g DM substrate incubated in reducing GHG production *in vitro*, particularly methane (CH4), from the diet of steers during anaerobic incubation in rumen fluid. Total gas production, CH4, CO, H2S, and fermentative characteristics were all measured *in vitro*.

**Results:**

Treatment AZI at a dose of 108 mg of liquid extract/g DM substrate produced the highest (*P* < 0.05) gas volume at 6 h, whereas CNA at a dose of 72 mg of liquid extract/ g DM substrate produced the least (*P* < 0.05) at 6 and 24 h, and Mix. at a dose of 72 mg of liquid extract/g DM substrate produced the least (P < 0.05) at 48 h. In addition, CH4 levels at 6 and 24 h of incubation (36 mg/g DM substrate) were highest (*P* < 0.05) for CNA, and lowest (*P* < 0.05) for AZI, whereas this variable was lowest (*P* < 0.05) at 72 mg of liquid extract for CNA at 24 and 48 h. At 6 and 24 h, CO volume was highest (*P* < 0.05) for AZI at 108 mg of liquid extract and lowest (*P* < 0.05) for Mix. at 72 mg of liquid extract. Treatment Mix. had a high (*P* < 0.05) concentration of short chain fatty acids at 72 mg of liquid extract/g DM of substrate.

**Discussion:**

In general, herbaceous perennial plants, such as AZI and CNA, could be considered suitable for mitigating enteric GHG emissions from animals. Specifically, the treatment Mix. achieved a greater sustainable reduction of 67.6% in CH4 and 47.5% in H2S production when compared to either AZI. This reduction in CH4 might suggest the potential of the combination of both plant extracts for mitigating the production of GHG from ruminants.

## Introduction

Enteric greenhouse gas (GHG) emissions have been a crucial ecological concern for humanity during the last two decades (1). From these emissions, ruminant livestock contributes the most, particularly beef and dairy cattle, about 7–18% of global GHG emissions, represented by methane (CH_4_), carbon dioxide (CO_2_), carbon monoxide (CO), hydrogen sulfide (H_2_S), and nitrogen oxide (2). In the United States, for example, livestock produced 3.10% of total GHG emissions in 2009, ranking second in terms of CH_4_ emissions and third in terms of non-CO_2_ emissions (3). These gases have a warming potential between 25 and 298 CO_2_ equivalent kg/kg (4). On-farm emissions account for a sizable portion of the carbon footprint associated with the dairy or beef supply chains (5, 6). Thus, ruminants and their associated gaseous emissions (enteric or manure) including CH_4_, CO_2_, and non-CO_2_ emissions should be the primary focus of any successful GHG mitigation effort. Additionally, in the rumen, H_2_S and CO originate from ruminal fermentation, and are utilized by the archaea in the synthesis of CH_4_, as detailed in Russel et al. (7). Components containing sulfur and sulfate can use H+ at low concentrations to produce H_2_S; therefore, interfering with methanogenesis CH_4_ (8, 9). Reducing sulfite to produce H_2_S consumes eight electrons and thus can be an alternate electron acceptor (8). On the other hand, CO may also interfere with CH_4_ formation because of its inhibitory effect on some ruminal bacteria (10). Therefore, evaluating the production of these gasses is important as well.

There are numerous strategies tested over the last few decades to reduce the GHG emissions from livestock production systems, including the use of natural products and their potential applications as feed additives. In this context, a variety of secondary metabolites are produced by plants as a means of protecting themselves from parasite attacks (11). These compounds include tannins, saponins, essential oils, cinnamic acids, flavonoids, and other phenolics, all of which have the potential to be used as anti-methanogenic chemicals (12). Notably, it has been demonstrated that supplementing with medicinal plant either as an extract or fermented has a beneficial effect on reducing methane production by altering the composition and structure of the ruminal microbial community (13–16). Indeed, by altering metabolic pathways, the addition of favorable additives to animal feed results in a subsequent reduction in the substrate availability for methanogenesis (17).

The use of fodder trees has been reported to improve ruminal degradation of nutrients, volatile fatty acids (18), microbial protein synthesis in the rumen (19), and reduce methanogens possibly due to the presence the secondary metabolites that may influence microbial activity (20, 21). In particular, trees such as *Azadirachta indica* (AZI), and *Cnidoscolus angustidens* (CNA) (22) may represent a good option to mitigate methanogenesis. One study use the *in vitro* fermentation technique to evaluate AZI and CNA in horse feces (18). It is worth noting, however, that studies conducted *in vitro* (23–25) should be confirmed by *in vivo* methods. Despite the promising effects of AZI and CNA for the mitigation of CH_4_ emission, to the authors’ knowledge, there is limited research evaluating their effects in the rumen (26), which represents a research gap that needs to be addressed in the scientific literature. In this regard, the combination of two or more plant extracts might provide unique biological characteristics that differ from the individual extracts because of the potential synergistic effect (27, 28). For example, certain phytogenic compound, such as the essential oils, possess a mixture of phytochemicals and their synergistic effects may lead to the synthesis of new compounds with different bioactivity (29). In particular, AZI is rich in essential oils, including 9.5% palmitic acid, 5.0% stearic acid, 32.0% oleic acid, 52.0% linoleic acid 0.51% linolenic acid (30), and other compounds such as azadirachtin, nimbolinin, nimbin, and quercetin (31). In the present experiment, our hypothesis was that the incubations with the extracts of AZI and CNA will effectively reduce the production of GHG under *in vitro* conditions. We also hypothesized that the combination of both treatments may result in a synergistic effect for the mitigation of GHG production.

Therefore, the objective of this study was to evaluate effects of aqueous leaf extracts of AZI, CNA or their combination on rumen GHG production *in vitro*.

## Materials and methods

### Collection of plants and preparation of extracts

The protocol of this *in vitro* study did not require the approval of the Animal Care and Use Committee because the utilized ruminal fluid was collected from slaughtered animals that were not part of the experiment.

Bioactive compounds are commonly present in the plants, but their concentration may vary according to plant maturity. For this reason, the vegetative material was collected throughout 7 months for both plants, when their phenological stage changes to flowering. In CNA the root was used, whereas in AZI the leaves were used. Roots of the CNA were taken manually from five plants in Puente de Ixtla, Morelos, Mexico, in the month of August 2021, later they were washed, peeled and frozen (approximately between –16°C and 24°C) for later use. Leaves samples of the AZI, were taken from five trees in the municipality of Tecpan de Galeana, Guerrero, Mexico, in February 2022, after which this vegetative material was dried at 40°C (BINDER Model FD115 Incubator, Germany) and ground (Willey Mill Model 5KH39ON5525, Mexico with a 1 mm sieve) in the bromatology laboratory of the Faculty of Veterinary Medicine and Animal Science of the Autonomous University of the State of Mexico. Finally, it was stored at room temperature (i.e., 25–30°C) for later use.

To prepare the extract of each plant species, we followed a protocol similar to reports from other researchers (18). Briefly, collected samples of individual species were ground into 1 mm length using a blender and subsequently extracted using 1 g of sample for every 8 mL of distilled water. Plant samples were soaked and incubated in water at 27–29°C for 72 h in closed 5 L glass containers. After incubation, the contents were filtered through 4–5 layers of gauze and the extract was collected. For practicality and because of the timeframe associated with the preparation of the leaf extracts, these were prepared weekly (at a stock volume of 8 L each). This mixture was stored at 2°C –4°C prior to use for the *in vitro* incubation, in order to avoid any further fermentation. Sometimes the extract was used immediately after preparation for the *in vitro* incubation, but no differences in fermentation performance was found between fresh and stored extract.

### Chemical analysis

Dietary substrate samples were analyzed in triplicate for DM, ash, organic matter (OM), nitrogen (N) and ether extract according to AOAC methods (32, 33). The analyses of neutral detergent fiber (NDFom) and acid detergent fiber (ADF) were performed in accordance with AOAC standards (32) using an ANKOM200 Fiber Analyzer Unit (ANKOM Technology Corp., Macedon, NY). The NDF was assayed without the use of alpha amylase, but with sodium sulphite in the NDF. Both NDF and ADF are expressed without residual ash as reported by others (34, 35).

### Phenolic content of extracts

Total phenolic content of the extracts were evaluated by a colorimetric method using Folin–Ciocalteu reagent, this method is based on a redox reaction mechanism where the phenolic compounds react with the folin-cicalteu reagent and give rise to a blue coloration, which is quantifiable in spectrophotometry at 765 nm based on a straight pattern of gallic acid. In brief, 1 mL of leaf extract was dissolved in 2 mL of methanol, and 500 μL aliquots of extract were combined with 2.5 mL Folin–Ciocalteu reagent (ten-fold diluted) and 2.5 mL (75 g/L) sodium carbonate. The tubes were vortexed for 10 s and allowed to stand at 25°C for 2 h. After 2 h of incubation at 25°C, absorbance at 765 nm was measured against a reagent blank. Total phenolic content was expressed as mg of gallic acid equivalent (GAE)/g. Total flavonoid content of the extracts was evaluated by using a modified AlCl_3_ calorimetric method (36). In a 10 mL volumetric flask, 1 mL of leaf extract was dissolved in 2 mL of methanol. A volume of each 200 μL leaf extract was filled to a prepared glass vial. Then, 75 μL of 5% NaNO_3_ was added to a prepared glass vial and abruptly sealed. The sealed glass vials were then stored at temperatures of 30–35°C for 5 min. Following that, each vial was added with 1.25 mL AlCl_3_ and 0.5 mL NaOH. The sealed glass vials were then sonicated and incubated for 5 min at a temperature of 30–35°C. Following incubation, the absorbance of all working and standard solutions was measured at 510 nm against a methanol blank. Estimation of flavonoid content of extracts was performed using the quercetin standard calibration curve, and the flavonoid content of the extracts was represented as μg of quercetin equivalent per one gram of dry extract.

### Rumen incubation

In the morning of each *in vitro* incubation day (06:00 h), rumen contents (2 kg/animal that included semisolid and liquid phases) were collected from four crossbreed steers (≈400 ± 20 kg body weight) for each incubation run. The ruminal contents of the four steers were collected immediately after slaughtering the animals in the city of Toluca, State of Mexico, and used as source of inocula for the *in vitro* incubations. Collection of the ruminal fluid is conducted as representative samples from different locations of the rumen (cranial, ventral, dorsal and caudal regions). After that, the collected ruminal contents were transferred immediately at the morning to the laboratory and mixed to get a pool rumen content sample, and then mixed with the Goering and Van Soest buffer solution (37) in a 1:4 vol/vol ratio (10 mL mixed rumen fluid and 40 mL of the buffer solution). Following that, the incubation media was subsequently mixed and strained through four layers of cheesecloth into a flask with an O_2_-free headspace and used to inoculate three replicates of incubation in 120-ml serum bottles containing 0.5 g substrate. The diet (substrate) was formulated according to NRC requirements for finishing cattle (the ingredients and chemical composition are shown in Table 1).

**Table 1:** Ingredient and chemical composition of the dietary substrate used for the *in vitro* fermentation.

Item	Content
Concentrate,^1^ g/kg DM	800
Alfalfa hay, g/kg DM	200
Chemical composition	
Organic matter, g/kg DM	870
Crude protein, g/kg DM	130
Non-fiber carbohydrates, g/kg DM	580
Crude fiber, g/kg DM	130
Ether extract, g/kg DM	30
Total phenols, mg gallic acid equivalent/g in *A. indica*	72.6
Flavonoids in the lyophilized extracts of *A. indica*, mg quercetin equivalent/g	122.2
Total phenols, mg gallic acid equivalent/g in *C. angustidens*	65.3
Flavonoids in the lyophilized extracts of *C. angustidens*, mg catechin equivalent/g	5.6

1 The concentrate was composed (g/kg DM) of 91 alfalfa hay, 180 wheat straw, 255 ground corn, 120 wheat bran, 163 gluten, 120 soybean meal, 70 molasse, 1 vitamins/minerals.

The study was conducted as a completely randomized experimental design replicated 3 times. The following treatments were added to the substrate in four doses of extract: 0, 36, 72 and 108 mg of liquid extract per gram of DM of substrate incubated of AZI, CNA or the mixture of the aqueous extracts (Mix. 1:1 vol/vol). Three vials were also included as blanks (the bottles containing rumen fluid and the *in vitro* buffer solution, but with no dietary substrate). There were 3 replicates in each group, and the dosages used were based on previous knowledge on the utilization on these extracts (18). However, dosages were adjusted for the incubation with ruminal fluid and utilizing cattle dietary substrates.

**Figure 1:**
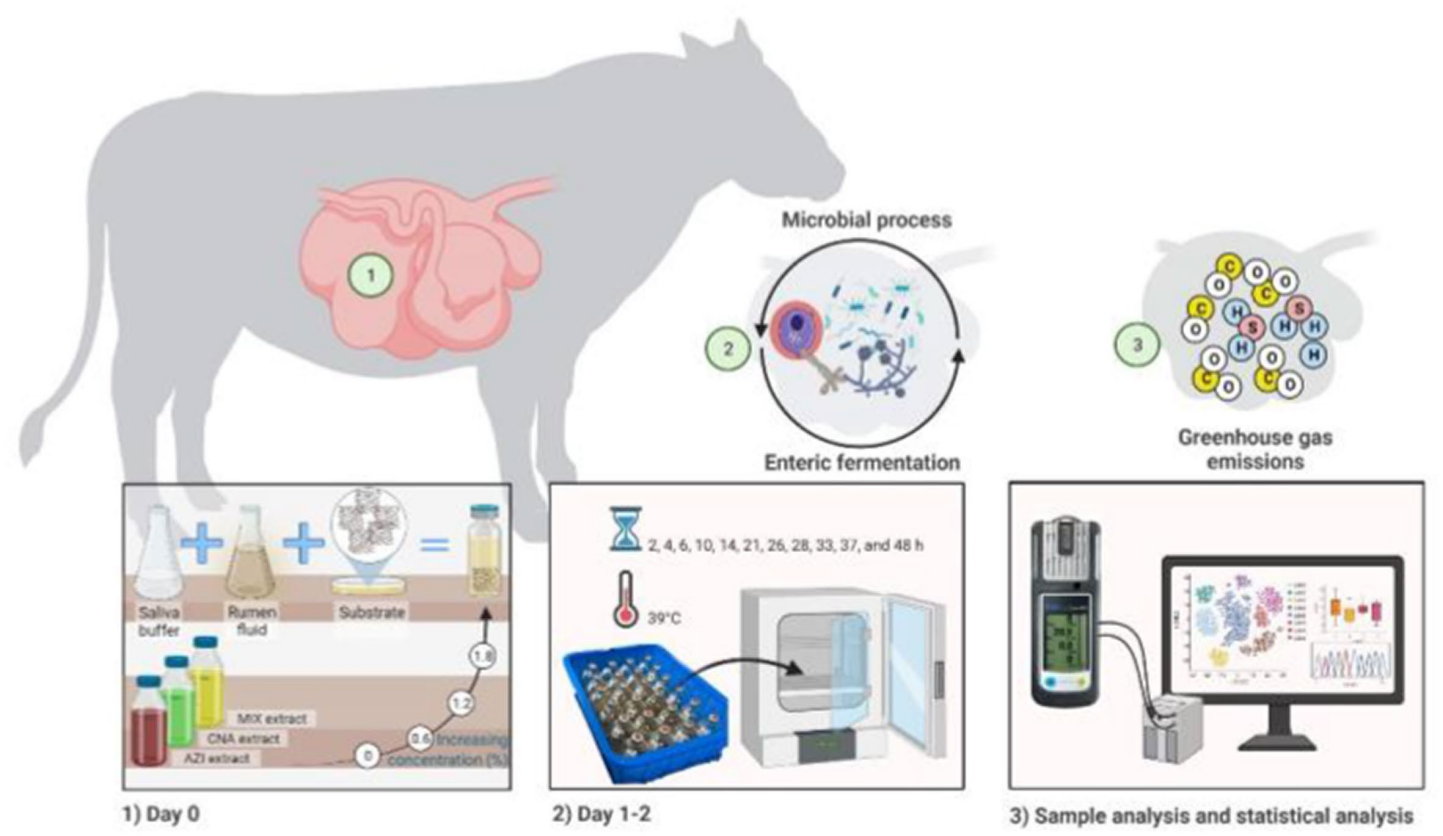
*In vitro* fermentation illustrating the synergistic role of treatment Mix consisting of *Azadirachta indica* and *Cnidoscolus angustidens* (1:1, AZI: CNA) at 72 mg extract/g DM of substrate as a feed additive contributes for reducing GHG as well as the other measured gases.

All assays (substrate, extracts doses, mixture of rumen liquor with Goering and Van Soest buffer solution) were mixed and prepared immediately before *in vitro* incubation. Three incubation runs were performed in 9 days (3 days for each run). A total of 135 bottles (3 bottles of each triplicate sample within each of the 3 plant species extracts) (2 species and 1 mixture of both), in 4 doses of extract for each plant species, in 3 runs on different weeks, with 9 bottles as blanks per each run (i.e., 3 rumen fluid only, 3 buffer only and 3 mixed rumen fluid with buffer only) were incubated for 48 h. Once all bottles were filled, the prepared bottles were flushed with CO_2_ and quickly closed with rubber stoppers, shaken and placed in an incubator at a temperature of 39°C. Total gas production (psi) was recorded at 2, 4, 6, 10, 14, 21, 26, 28, 33, 37, and 48 h utilizing pressure transducer (Extech Instruments, Waltham, United States) following the technique of Theodorou et al. (38). These measurement timepoints allowed evaluation of the asymptotic gas production, the rate of gas production as well as the lag time before gas production begins. The productions of CH_4_, CO, and H_2_S were also measured at the same hours of incubation using a diffusion-based gas detector (MONITOR de Dräger Safety X-am 2,500, Lübeck, Germany) using a sample of 5 mL. After each value was recorded, using a syringe needle, the gas was dispersed to avoid gas accumulation (Figure 1). The device used consists of a portable gas detection instrument for monitoring the concentration of a variety of gases (i.e., CH_4_, CO, and H_2_S). With this method, after calibration has been performed, the sample of gas (5 mL) is collected and inserted into the instrument. Then the output results are visualized in a matrix display; data may also be downloaded in a computer for better visualization of the results. This approach for the measurement of gasses has been previously tested and its use has been reported elsewhere (39, 40).

### Dry matter degradation

Measurement of DM degradation was performed following the approach used by Rodriguez et al. (34). Briefly, after 48 h of incubation, bottles were uncapped and the pH was measured using a digital pH meter (Conductronic pH15, Puebla, Mexico), and the residual of each bottle was filtered through Whatman filter paper, and the bottles were rinsed thoroughly in order to collect the feed residues, similar to the approach used by Zhang et al. (41). After fermentation, residues were dried at 45°C for 72 h to estimate dry matter degradability [DMD; (37)]. The weight loss after drying was used to calculate undegradable DM. At 48 h of incubation, the DM degradability of the substrate (i.e., apparent degradation) was determined as the difference between the substrate’s DM content and its undegradable DM (34).

### Calculation and statistical analysis

To estimate the kinetic parameters of gas production (GP), CH_4_, CO, and H_2_S, the results of GP, CH_4_, CO, and H_2_S (mL/g DM) were fitted using the NLIN option of SAS 9.4 to the France, Dijkstra model (42). The model is shown in the following equation:


(1) $$A=b\times \left(1-e^{-c\left(t-\text{lag}\right)}\right)$$

where A is the volume of GP, CH_4_, CO and H_2_S at time t; b the asymptotic GP, CH_4_, CO, and H_2_S (mL/g DM); c is the rate of GP, CH_4_, CO, and H_2_S (/h), and lag (h) is the discrete lag time is the time prior to production of gas (GP), CH_4_, CO, and H_2_S.

Metabolizable energy (ME, MJ/kg DM) was estimated as stated by Menke et al. (25) equation as:


(2) ME=2.20+0.136GP+0.0057CP(gkgDM)

*In vitro* organic matter digestibility (OMD, g/kg OM) according to Menke and Steingass (25). The aquation was: OMD (%) = 16.49 + 0.9042 × GP + 0.0492 × CP + 0.0387 × ash.

Short chain fatty acids concentration (SCFA) was calculated according to Getachew, Makkar equation (43). The calculations are illustrated in the following equation:


(3) SCFA(mmol200mgDM)=0.0222GP−0.00425

where GP is the 24 h net gas production (mL/200 mg DM).

Experimental design was completely randomized with repeated measures over time. Before performing the statistical analyses, data from each of the three runs within the same sample of each of the three individual samples (AZI, CNA, and Mix.) were averaged, and the mean values of each individual sample were used as the experimental unit. Results of rumen fermentation parameters, CH_4_ conversion efficiency were analyzed as a factorial experiment using the PROC GLM option of SAS 9.4 equation as:


(4) $$Y_{\text{ijk}}=\mu +P_{i}+E_{j}+{\left(P\times D\right)}_{\text{ij}}+\varepsilon \text{ijk}$$

where Y_ij_ is every observation of the *i* plant species (Pi) with *j* extract dose (E_j_); μ is the general mean; (P × E)_ij_ is the interaction between plant species and extract dose; ɛijk represents the experimental error, normally distributed with the average 0 and the constant variance. Linear and quadratic polynomial contrasts were used to examine responses of greenhouse gas production and gas production of the fermented diet fermentation with the increasing addition levels (doses) of the plant species extracts. Statistical significance was declared at *p* < 0.05, and tendency was discussed if *p* > 0.05 and ≤ 0.10.

## Results

### Production of total Gas, CH_4_, CO, and H_2_S

**Figure 2:**
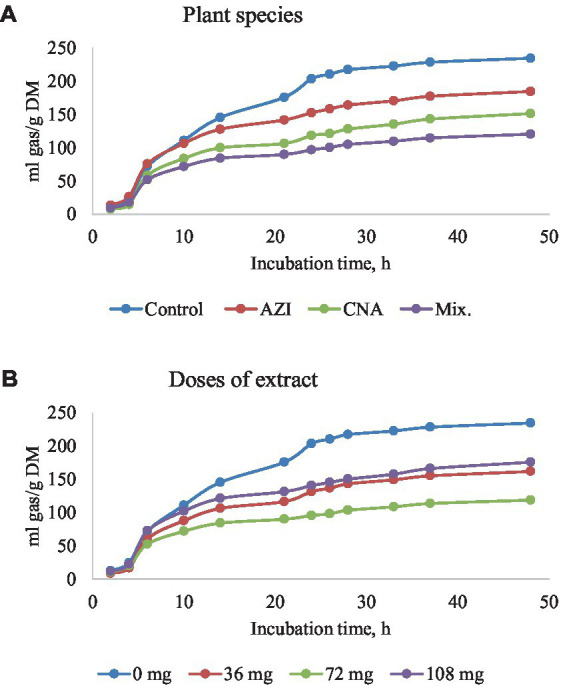
Rumen total gas production [mL/g dry matter (DM)] at different hours of incubation as affected by the dietary inclusion with the aqueous extract of *A. indica* (AZI)*, C. angustidens* (CNA) or their combination (Mix. 1:1, vol/vol) – **(A)** at 0, 36, 72 and 108 mg of liquid extract/g DM **(B)** incubated with rumen contents slaughtered steers.

**Figure 3:**
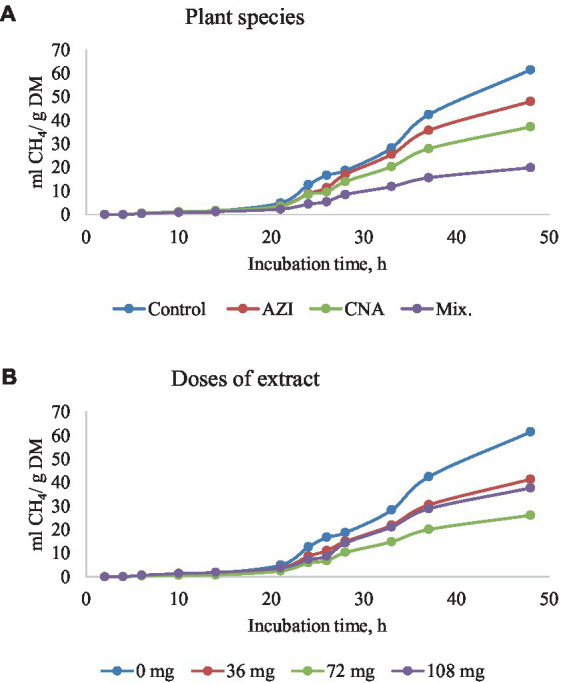
Rumen methane (CH_4_) production [mL/g dry matter (DM)] at different hours of incubation as affected by the dietary inclusion with the aqueous extract of *A. indica* (AZI)*, C. angustidens* (CNA) or their combination (Mix. 1:1, vol/vol) – **(A)** at 0, 36, 72 and 108 mg of liquid extract/g DM **(B)** incubated with rumen contents slaughtered steers.

**Figure 4:**
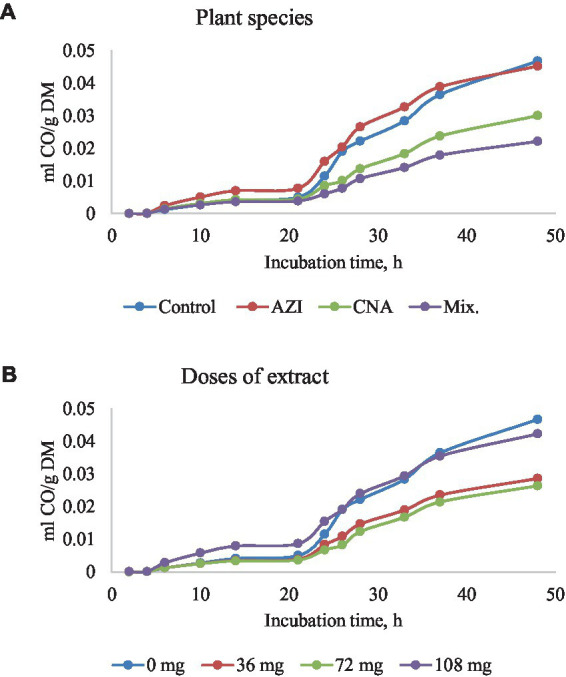
Rumen carbon monoxide (CO) production [mL/g dry matter (DM)] at different hours of incubation as affected by the dietary inclusion with the aqueous extract of *A. indica* (AZI)*, C. angustidens* (CNA) or their combination (Mix. 1:1, vol/vol) – **(A)** at 0, 36, 72 and 108 mg of liquid extract/g DM **(B)** incubated with rumen contents slaughtered steers.

**Figure 5:**
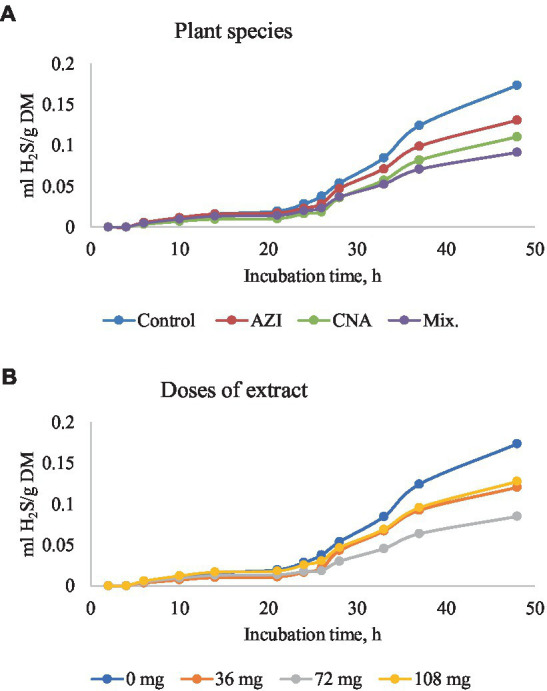
Rumen hydrogen sulfide (H_2_S) production [mL/g dry matter (DM)] at different hours of incubation as affected by the dietary inclusion with the aqueous extract of *A. indica* (AZI), *C. angustidens* (CNA) or their combination (Mix. 1:1, vol/vol) – **(A)** at 0, 36, 72 and 108 mg of liquid extract/g DM **(B)** incubated with rumen contents slaughtered steers.

The mixture of the leaf extracts (Mix.) resulted in the lowest (*p* < 0.05) gas produced for plant species for all the gases determined, while the dose extract at 72 mg of either AZI, CNA or Mix. produced the least (*p* < 0.05) gas over the 48-h incubation period (Figures 2–5A,B).

The production of gas at time points 6, 24 and 48 h are shown for practicality, and because these are key timepoints that allow illustration of the fermentation kinetics throughout the evaluated fermentation timeframe. There was a significant difference on the parameters measured. An increase in period of *in vitro* fermentation (hour of incubation) led to an increase (*p* < 0.05) in gas produced. Increases in dosage and incubation elicited an increased (*p* < 0.05) volume of gas for AZI while CNA and Mix recorded high volumes at a low dose (36 mg) after 48; these results are shown in Table 2. We also found a quadratic effect (*p* < 0.05) of CNA on the asymptotic gas production and lag with the lowest values for these variables observed for the dose 72 mg/g fermentation substrate. Likewise, the asymptotic gas production was quadratically affected by Mix., with the lowest (*p* < 0.05) value observed for dosage 72 mg/g of fermentation substrate.

**Table 2:** *In vitro* rumen gas production kinetics and total production at 6, 24, and 48 h of the incubated diet with the leaf aqueous extract of *A. indica* (AZI), *C. angustidens* (CNA) or their combination (Mix. 1:1, vol/vol) at 0, 36, 72 and 108 mg liquid extract/g DM incubated with rumen fluid of slaughtered steers.

Plant species extract	Extract dose (mg/g DM)	Gas production kinetics^1^			Gas production (mL gas/g DM incubated)		
		b	c	*Lag*	6	24	48
AZI	0	224	0.05	1.15	71.9	203	234
	36	148	0.06	2.01	56.1	129	158
	72	167	0.07	1.42	80.0	150	186
	108	190	0.07	1.05	90.2	177	209
	Linear	0.4415	0.2059	0.8993	0.1454	0.5307	0.5626
	Quadratic	0.3004	0.5993	0.6463	0.9214	0.2743	0.3563
CNA	0	224	0.05	1.15	71.9	203	234
	36	170	0.06	1.14	66.1	151	184
	72	88.1	0.04	5.40	37.8	66.5	87.0
	108	168	0.08	2.94	71.2	136	182
	Linear	0.1495	0.311	0.2071	0.9674	0.0634	0.2324
	Quadratic	0.0068	0.4169	0.0179	0.0353	0.0051	0.0088
Mix	0	224	0.05	1.15	71.9	203	234
	36	127	0.08	1.66	59.5	113	142
	72	93.4	0.07	1.38	39.6	69.1	82.5
	108	124	0.07	0.86	56.0	107	135
	Linear	0.0027	0.5346	0.598	0.3639	0.0082	0.011
	Quadratic	0.0039	0.5606	0.4357	0.1261	0.0067	0.0042
SEM pooled^2^		21.1	0.015	0.488	8.9	20.3	23.9
*p*-value							
Extract		0.0785	0.7142	0.0109	0.0600	0.0558	0.0571
Dose		0.0001	0.4229	0.0423	0.0744	<0.0001	0.0002
Linear		0.0038	0.1067	0.3923	0.9423	0.0030	0.0129
Quadratic		0.0001	0.9539	0.0076	0.0124	0.0001	0.0001
Extract × Dose		0.3705	0.8647	0.0127	0.1564	0.2960	0.2696

1 b is the asymptotic gas production (mL/g DM); c is the rate of gas production (/h); lag is the initial delay before gas production begins (h).

2 SEM standard error of the mean.

Similar to total gas, increased dosage resulted in increased (*p* < 0.05) CH_4_ gas for AZI while the opposite was observed in Mix. The highest (*p* < 0.05) CH_4_ was recorded for CNA at 36 mg dose. There was no significant difference for CH_4_ proportions when AZI increased with an increase in dosage. After 48 h, CO volume was high (*p* < 0.05) for AZI at 108 mg dose and least (*p* < 0.05) was seen for Mix. at 72 mg dose after 48 h. After 6 h, there was no H_2_S gas produced for all the plant species. High (*p* < 0.05) volume was observed for AZI after 48 h at a 108 mg dose (Tables 3–5).

**Table 3:** *In vitro* rumen methane (CH_4_) production at 6, 24, and 48 h of the incubated diet with the leaf aqueous extract of *A. indica* (AZI), *C. angustidens* (CNA) or their combination (Mix. 1:1, vol/vol) at 0, 36, 72 and 108 mg of liquid extract/ g DM incubated with rumen fluid of slaughtered steers.

Plant species extract	Extract dose (mg/g DM)	CH_4_ production kinetics^1^			CH_4_ production (ml CH_4_/g DM incubated)			CH_4_ (mL CH_4_/100 mL gas)		
		b	c	*Lag*	6	24	48	6	24	48
AZI	0	16.2	0.02	17.4	0.44	12.6	61.3	0.61	6.21	26.1
	36	18.9	0.03	17.0	0.17	8.13	42.8	0.30	6.30	27.1
	72	16.5	0.03	16.2	0.29	10.1	45.5	0.36	6.70	24.4
	108	18.2	0.03	13.9	0.75	8.16	55.4	0.83	4.59	26.5
	Linear	0.5753	0.3944	0.0191	0.2355	0.4362	0.7142	0.3535	0.5632	0.920
	Quadratic	0.8121	0.3291	0.5891	0.1886	0.9531	0.368	0.1613	0.4579	0.8214
CNA	0	16.2	0.02	17.4	0.44	12.6	61.3	0.61	6.21	26.1
	36	25.7	0.02	14.9	0.77	12.9	58.1	1.16	8.55	31.5
	72	30.6	0.04	17.1	0.23	2.41	12.1	0.61	3.62	13.9
	108	18.6	0.03	15.7	0.66	10.2	41.2	0.93	7.55	22.6
	Linear	0.8444	0.4948	0.5115	0.3126	0.6481	0.0704	0.2295	0.6181	0.2476
	Quadratic	0.22	0.1936	0.7994	0.1178	0.0681	0.0016	0.8428	0.3425	0.0583
Mix	0	16.2	0.02	17.4	0.44	12.6	61.3	0.61	6.21	26.1
	36	27.3	0.02	13.9	0.57	5.00	23.0	0.96	4.41	16.2
	72	31.6	0.03	15.6	0.30	5.06	20.4	0.76	7.32	24.7
	108	51.0	0.04	17.2	0.46	3.19	16.2	0.82	2.96	11.9
	Linear	0.0436	0.174	0.9161	0.9514	0.0651	0.0011	0.5099	0.305	0.1491
	Quadratic	0.8772	0.9592	0.2681	0.5961	0.4776	0.0491	0.7009	0.3684	0.2751
SEM pooled^2^		5.852	0.0074	1.1394	0.148	3.119	7.491	0.832	1.044	3.468
*p*-value										
Extract		0.0496	0.8704	0.958	0.6483	0.3459	0.0056	0.0708	0.4763	0.3551
Dose		0.2218	0.1856	0.2201	0.1510	0.1284	0.0003	0.3250	0.9179	0.0255
Linear		0.0490	0.1024	0.1022	0.2209	0.0706	0.0019	0.1086	0.5825	0.1068
Quadratic		0.5242	0.1635	0.8449	0.0571	0.1147	0.0006	0.5691	0.8110	0.6363
Extract × Dose		0.2641	0.8417	0.3410	0.4051	0.5507	0.0400	0.3855	0.4401	0.0411

1 b is the asymptotic CH_4_ production (ml/g DM); c is the rate of CH_4_ production (/h); lag is the initial delay before CH_4_ production begins (h).

2 SEM standard error of the mean.

**Table 4:** *In vitro r*umen carbon monoxide (CO) production at 6, 24, and 48 h of the incubated diet with the leaf aqueous extract of *A. indica* (AZI), *C. angustidens* (CNA) or their combination (Mix. 1:1, vol/vol) at 0, 36, 72 and 108 mg of liquid extract/ g DM incubated with rumen fluid of slaughtered steers.

Plant species extract	Extract dose (mg/g DM)	CO production kinetics^1^			CO production (mL CO/g DM incubated)		
		b	c	*Lag*	6	24	48
AZI	0	124	0.31	3.98	0.001	0.013	0.224
	36	76.9	0.03	7.53	0.001	0.006	0.025
	72	120	0.03	3.77	0.002	0.012	0.043
	108	154	0.03	4.55	0.005	0.031	0.068
	Linear	0.2506	0.1998	0.7492	0.0236	0.0905	0.2512
	Quadratic	0.3904	0.4483	0.7511	0.3142	0.2669	0.3753
CNA	0	124	0.31	3.98	0.001	0.013	0.224
	36	107	0.03	5.69	0.002	0.012	0.038
	72	128	0.02	8.37	0.001	0.005	0.020
	108	116	0.02	3.69	0.002	0.009	0.033
	Linear	0.8341	0.1873	0.8825	0.0667	0.1992	0.1667
	Quadratic	0.809	0.4303	0.0232	0.2555	0.0416	0.3478
Mix	0	124	0.31	3.98	0.001	0.013	0.224
	36	99.1	0.02	4.81	0.001	0.007	0.023
	72	128	0.02	7.66	0.001	0.004	0.016
	108	112	0.02	7.90	0.002	0.007	0.027
	Linear	0.7456	0.1928	0.0458	0.3466	0.029	0.1548
	Quadratic	0.7403	0.4213	0.265	0.5796	0.0135	0.3429
SEM pooled^2^		19.841	0.0739	1.1269	0.0004	0.0024	0.0511
*p*-value							
Extract		0.9803	0.9971	0.4463	0.1441	0.0383	0.9614
Dose		0.2398	0.0501	0.0856	0.0083	0.0608	0.0311
Linear		0.8484	0.0215	0.1787	0.0022	0.4134	0.0197
Quadratic		0.9825	0.1658	0.0382	0.1425	0.0206	0.1021
Extract × Dose		0.7916	1.0000	0.0475	0.1108	0.0307	1.0000

1 b is the asymptotic carbon monoxide (CO) production (ppm); c is the rate CO production (/h); Lag is the initial delay before CO production begins (h).

2 SEM standard error of the mean.

**Table 5:** *In vitro* rumen hydrogen sulfide (H_2_S) production at 24, and 48 h of the incubated diet with the leaf aqueous extract of *A. indica* (AZI), *C. angustidens* (CNA) or their combination (Mix. 1:1, vol/vol) at 0, 36, 72 and 108 mg of liquid extract/g DM incubated with rumen fluid of slaughtered steers.

Plant species extract	Extract dose (mg/g DM)	H_2_S production kinetics^1^			H_2_S production (mL/g DM incubated)^2^	
		b	c	*Lag*	24	48
AZI	0	2,207	0.02	3.72	0.02	0.17
	36	973.1	0.02	5.60	0.01	0.12
	72	3,246	0.01	4.26	0.02	0.11
	108	1,410	0.02	5.22	0.02	0.15
	Linear	0.7271	0.9633	0.5148	0.971	0.6401
	Quadratic	0.4734	0.8114	0.9143	0.7534	0.2094
CNA	0	2,207	0.02	3.72	0.02	0.17
	36	448.6	0.03	3.26	0.01	0.13
	72	1,087	0.02	6.88	0.01	0.07
	108	858.4	0.02	7.21	0.02	0.12
	Linear	0.0967	1.0000	0.0457	0.6826	0.1242
	Quadratic	0.4937	0.7985	0.3008	0.1249	0.0147
Mix	0	2,207	0.02	3.72	0.02	0.17
	36	570.4	0.02	2.70	0.02	0.10
	72	592.6	0.02	5.05	0.01	0.06
	108	543.1	0.02	5.63	0.02	0.10
	Linear	0.037	0.5641	0.4067	0.6638	0.0195
	Quadratic	0.2118	0.588	0.8467	0.2873	0.0083
SEM pooled^3^		675.90	0.0053	1.2974	0.0069	0.0175
*p*-value						
Extract		0.3395	0.6399	0.6107	0.6335	0.2013
Dose		0.2385	0.2128	0.1438	0.1319	0.0008
Linear		0.1273	0.6775	0.0558	0.6082	0.0195
Quadratic		0.9212	0.7028	0.6002	0.0716	0.0004
Extract × Dose		0.8723	0.7548	0.6332	0.8439	0.8479

1 b is the asymptotic hydrogen sulfide (H_2_S) production (ppm); c is the rate of H_2_S production (/h); Lag is the initial delay before H_2_S production begins (h).

2 H_2_S production was not detected at 6 h of incubation.

3 SEM standard error of the mean.

### Fermentation characteristics and CH_4_ conversion efficiency

The ruminal pH tended to decrease (*p* = 0.09) for AZI and decreased (*p* < 0.01) for CNA plant species as dose levels were increased. A similar trend was observed for DMD and ME in the presence of CNA. There was a significant difference (*p* < 0.05) at dose level for DMD and ME (Table 6). Additionally, OMD content was 67.8%. However, CH_4_ conversion efficiency to SCFA, ME or OM were not affected by AZI, CNA or Mix any supplemented dose levels (*p* ≥ 0.12).

**Table 6:** *In vitro r*umen fermentation profile and methane conversion efficiency to short chain fatty acids (CH_4_: SCFA at 24 h, mmol/mmol), metabolizable energy [CH_4_: ME (g/MJ)], and organic matter [CH_4_: OM (ml/g)] of the dietary inclusion with different doses of *A. indica* (AZI), *C. angustidens* (CNA) or their combination (Mix. 1:1, vol/vol) at 0, 36, 72 and 108 mg of liquid extract/g DM incubated with rumen fluid of slaughtered steer.

Plant species extract	Extract dose (mg/g DM)	Rumen fermentation profile^1^				Methane conversion efficiency		
		pH	DMD%	SCFA mmol/g DM	ME, MJ/kg DM 24 h	CH_4_: ME (g/MJ)	CH_4_:OM (mL/g)	CH_4_: SCFA at 24 h (mmol/mmol)
AZI	0	6.94	62.0	29.0	8.61	39.3	6.70	14.0
	36	6.97	57.5	34.6	6.91	35.6	4.88	9.04
	72	6.90	56.9	35.6	7.40	45.4	6.44	11.2
	108	6.85	58.3	30.3	8.02	29.5	4.62	9.06
	Linear	0.0971	0.5307	0.2941	0.5306	0.5638	0.4818	0.4362
	Quadratic	0.8716	0.2743	0.0002	0.2744	0.4563	0.7589	0.9531
CNA	0	6.94	62.0	29.0	8.61	39.3	6.70	14.0
	36	6.95	65.1	27.8	7.43	59.2	8.29	14.4
	72	6.67	62.7	28.9	5.48	26.5	1.81	2.67
	108	6.50	63.1	29.4	7.07	50.4	6.80	11.4
	Linear	0.0019	0.0634	0.7909	0.0635	0.6168	0.9754	0.6482
	Quadratic	0.5267	0.0051	0.7951	0.0051	0.3513	0.0919	0.0681
Mix	0	6.94	62.0	29.0	8.61	39.3	6.70	14.0
	36	6.56	60.6	34.3	6.55	25.9	3.37	5.55
	72	6.81	59.3	37.7	5.54	44.3	3.98	5.62
	108	6.81	66.5	27.8	6.42	20.3	2.31	3.54
	Linear	0.3347	0.0082	0.7655	0.0082	0.307	0.1168	0.0651
	Quadratic	0.5501	0.0067	0.0282	0.0067	0.3623	0.8142	0.4776
SEM pooled^2^		0.052	9.041	1.356	0.464	11.858	1.772	3.466
*p*-value								
Extract		0.0063	0.0558	0.0146	0.0559	0.4749	0.3813	0.3459
Dose		0.0050	<0.0001	0.0045	<0.0001	0.9175	0.3925	0.1284
Linear		0.0005	0.0030	0.9313	0.0030	0.5860	0.1967	0.0706
Quadratic		0.4233	0.0001	0.0007	0.0001	0.7955	0.2715	0.1147
Extract × Dose		0.0007	0.2960	0.0808	0.2961	0.4452	0.3826	0.5507

1 SCFA is the short chain fatty acids (mmol/g DM); DMD is the in vitro dry matter digestibility (%); ME is the metabolizable energy (MJ/kg DM).

2 SEM standard error of the mean.

## Discussion

To the authors’ knowledge, this is the first study evaluating the efficacy of leaf extracts from AZI and CNA, or their combination (Mix. 1:1) in a formulated ruminant diet as a viable alternative for reducing GHG production under *in vitro* conditions. Previous investigations have included these fodder trees in ruminant diets to evaluate whether the presence of tannins and/or saponin improves animal performance (20, 44), except for enteric CH_4_ and other gasses produced in the rumen that may influence the production of GHG as well as other gases produced within the rumen such as CO and H_2_S. However, the effects observed in these studies cannot be attributed solely to condensed tannins and/or saponin due to the synergistic and antagonistic effects of complex matrices such as flavonoids and other phenolics from AZI (45) and CNA (46). Several previous studies have examined synergistic compounds in animal nutrition and physiology (44–46). However, none of these compounds were derived from AZI and CNA as in the present experiment.

In addition to the total phenols and flavonoids that were measured in the plants extract, previous researchers have reported the presence of essential oils including palmitic acid, oleic acid and linoleic acid in CNA (30) as well as azadirachtin, nimbolinin, nimbin, nimbidin, nimbidol, sodium nimbinate, gedunin, salannin, and quercetin in AZI (31). Some of these compounds have been shown to have potent antibacterial properties (31), which may have influenced the rumen bacterial community profile and activity when used in the present study. Therefore, it is possible that the chemical compounds of the extracts found in this study in combination with those previously reported by other investigators acted synergistically enhancing their effect on the ruminal microbial fermentation dynamics, which results in an increased effect for reducing the synthesis the GHG as shown by the results from this experiment.

### Total gas production

Given the current global problem associated with GHG emissions, there is a growing interest in finding effective strategies to mitigate the emissions of enteric GHG from ruminants. Despite extensive research in this topic, mitigation strategies are still needed. According to our hypothesis, results from this study showed promising effects of two plant species for the mitigation of total gas production as well as GHG when included as aqueous extracts in ruminant diets *in vitro*.

Gas produced during *in vitro* fermentation reflects the extent of feed fermentation and digestion by the microbial community. Our findings indicated that insoluble feed substrate (*b*, asymptotic fraction) and total gas production showed a quadratic response, with these variables being reduced in the two evaluated fodder trees only when the dosages of extracts were 72 mg of liquid extract/g DM substrate. These quadratic responses in asymptotic GP with increasing levels of treatment Mix., which included the mixture of the two evaluated fodder trees suggest a potential antagonistic effect of the dose 72 mg/g DM substrate. However, greater dosages lead to a possibly synergistic effect when the plant extracts were combined. It is reasonable to assume that changes in metabolic processes in the rumen are dose-dependent. Thus, leaf extracts used in the present experiment might have an effect on the cumulative gas production after 48 h of *in vitro* incubation. Previously, the increase in gas volumes observed in extracts rich in condensed tannins, phenolics, and flavonoids could be attributed to improved rumen fermentation and nutrient digestibility (26). The latter study utilized an extract from *Cnidoscolus aconitifolius*, which was included in the ration in pelleted form and contained 2.3% of total tannins and 7.3% of total flavonoids; authors concluded that the use of these inclusion levels represents a promising approach to enhance rumen fermentation and to mitigate CH_4_ production. However, in the present study, AZI contained twofold the number of flavonoids (expressed as quercetin) that Totakul and coworkers used (26). This indicates that the increase in gas production can be attributed to the quercetin’s stimulatory effect on the ruminal microbes, resulting in increased activity (2). Furthermore, it has been asserted that diets containing tannins with low astringency could not negatively affect feed intake and animal growth performance, resulting in an increase in gas production as seen in AZI tree species, as reported by Waghorn (19). Previous research has demonstrated that synergistic compounds expressed as gallic acid (tannins) increase gas production as well as the dosage of tannins or flavonoids (47, 48). Thereby stimulating the growth of ruminal cellulolytic bacteria capable of fermenting organic substrates. In addition, a decrease in gas produced might be caused by the inhibitory effects of synergistic compounds such as the presence of AZI or CNA on methanogens during methanogenesis, as will be discussed further below. Therefore, results suggest that in the Mix. treatment, the combination of extracts may well have enhanced the potency of phytochemicals or synergistic compounds that each plant species contributed to the mixture, which may have resulted in the generation of new compounds that mitigated the activity of methanogens, thereby reducing the amount of total gas produced (2).

The significant increase in the concentration of individual gases after 20 h of incubation may be related to ruminal microbial dynamics. At the start of the *in vitro* fermentation, there is a period of time where the microbial community needs to adapt to the fermentation conditions. During this initial stage, feed degradation is minimal. However, as fermentation time increases, the microbial community completely adapts to the environment, there is increased degradation of feed as well as generation of soluble nutrients, which are taken up and fermented by the microbial community. Thus, this results in a significant increase in the concentration of individual gases, as observed in this study because fermentation and gas production are highly dependent on the availability of nutrients from substrates. However, it should be noted that the digestibility of the starch in substrates was not measured in this study. Reports have shown that starch availability for microbial fermentation may be affected not only by the type of corn processing (49), but also by several factors within a specific type of grain processing (50).

### CH_4_ production

The initial delay before CH_4_ production (*Lag*) decreased with increasing dose levels and CH_4_ gas production for AZI, whereas the opposite happened for Mix. treatment. As demonstrated in the AZI plant species extract, the delay would have been reduced as a result of the extract’s high dose providing a favorable environment for methanogens to thrive, resulting in high CH_4_ production. On the other hand, it is reasonable to assume that a synergistic effect among phytogenic compounds in Mix. treatment, which resulted in the inhibition of methanogens from the start of the *in vitro* fermentation, leading to an increase of the lag phase. On average, Mix. plant species achieved a greater sustainable reduction in CH_4_ production (approximately 67.58%). However, the proportion of CH_4_ was not affected by dietary substrate, and this may be due to a uniform decrease in the production of other gases, so that the proportion of CH_4_ was maintained. When protozoans are reduced, methanogens are suppressed as well because they have a symbiotic relationship in the rumen, as explained by Park and Yu (51). The quadratic effect of CNA on either total gas production or CH_4_ production suggests that if using CNA alone, the dosage should be 72 mg/g DM (not greater or lower) in order to achieve the most beneficial effect in the mitigation of GHG production. Synergistic chemicals have been shown to reduce CH_4_ production more effectively (47, 48). The synergistic chemicals have been shown to have an antibacterial impact in rumen methanogenesis by interfering with gram-positive bacteria’s outer membrane (23, 52) which may have occurred with the Mix. treatment used in the present experiment. Specifically, bacteria lose control of ion gradients, electron mobilization, phosphorylation cascades, protein translocation, and other enzymatic processes as a result of this motion (53). According to Seradj, supplementation with the phytonutrient Bioflavex, which is based on flavonoids, reduced CH_4_ emissions (54). Though these bioactive compounds have direct effects on methanogens by suppressing CH_4_ production, as seen in Mix. plant species, this improves animal productivity (21, 55). However, because of the nature of this *in vitro* study, the effects of the evaluated treatments on animal production performance could not be determined, and this is worth considering in future experiments.

### Carbon monoxide production and hydrogen sulfide production

To the author’s knowledge, the current *in vitro* results are the first to report on the incubation of AZI, CNA, or Mix. form, and the data indicate that incubating AZI, CNA, or Mix. form resulted in a longer delay before CO production (*Lag*), leading to reduced gas volumes, particularly at 7 h and above. This may also be another effect of a potential synergistic effect of the phytochemical compounds originating from the plants extract combined in Mix., which may have results in mitigation of ruminal methanogens. Thus, findings from the present study showing a reduction in CO production is consistent with the reports of Parra-Garcia et al. (56). Therefore, it can be concluded that CH_4_ and CO are positively related (57).

Extract of Mix. had a mitigation extent of approximately 47.51% on average, compared to CNA and AZI (36.59 and 24.71%, respectively). Indeed, the reduction in H_2_S gas was 62.07% at 72 mg Mix. Gerber et al. stated that the benefits of tannins consumption corresponded to reductions in N_2_O emissions and may have had an effect on H_2_S production (2). The H_2_ required for the formation of CH_4_ and H_2_S could have been used by other microbes or disposed in a sink (2, 58). Therefore, our results demonstrate a synergistic effect of the combination of phytochemicals in Mix. not only on GHG production, but also in associated molecules like H_2_S. However, given the wide range of compounds present in each plant species used in this study, further research is warranted to elucidate which specific combination of phytochemical results in the mitigation of methanogens and their byproducts during fermentation.

### Fermentation characteristics and CH_4_ conversion efficiency

The ruminal pH ranged between 6.50 and 6.97, which is normal for rumen ecology and the efficiency of rumen fermentation (26, 47). A greater production of short chain fatty acids (SCFAs) is most likely explained by rumen digestibility when the rumen fluids were incubated with Mix. The present results could be corroborating to previous observations that higher level of dietary CP and supplementation of CNA pellets resulted in increased gas production and *in vitro* digestibility (26, 59). In agreement with this report, a greater DMD and GP had been observed to linearly increase of availability CP in diet (60). This is in agreement with that of AZI in this study where its gas volumes increased with increase in DMD and could be attributed to the concentrate and AZI extracts, which might have enhanced the nutrient digestibility in the rumen (61). Additionally, synergistic compounds such as tannins, phenolic acids, and flavonoids were shown to have a greater stimulatory effect on DMD and OMD, implying an increase in SCFAs and a more acidic environment (47, 48). Findings of increased DMD and ME in AZI plant species appeared to be due to increased bacterial activity (62). The low digestibility observed for the dietary substrates could be due to a slow activity of the microbiome on the substrates during the initial stage of the *in vitro* incubation timeframe. The measurement of DMD at a longer incubation time point when microbial activity has increased may result in greater digestibility. Values of ME obtained in this study were greater than those reported by Njidda for eight browse leaves (63). These values ranged between 3.28 and 3.84 MJ/Kg. Though gas volumes were reduced, no effect on SCFAs was observed for all plant species. According to Salem et al., phytochemicals could have an effect on rumen modification by stimulating ruminal fermentation and increasing protein metabolism and digestibility of plant cell wall constituents (64). Regrettably, neither the microbial community profile nor the SCFA fraction including protein utilization were quantified in the current *in vitro* study, and thus the current results do not provide additional information to speculate on the fate of rumen fermentation. Therefore, additional research is necessary to shed further light on this subject. However, this study serves as a first approach to evaluate the effects of the plant extracts on GHG, which will be the basis for future experiments where we will include the evaluation of the microbial community (protozoa, bacteria and potentially the archaeal community) as well as the profile of volatile fatty acids.

The discrepancies between the results from different research settings may be due to a multitude of factors, including the composition of the non-tannic-supplemented diet utilized in the study, fermentation substrates, flavonoid bioavailability, and animal physiological states. The current observation of increased mitigation of sustainable rumen greenhouse gas emissions (primarily enteric CH_4_) under *in vitro* conditions appeared to validate our hypothesis. Overall, it suggested that the presence of flavonoids either in the form of AZI, CNA, or Mix. modulates the activity of flavonoids and other phytochemicals, in the *in vitro* fermentation of a ruminant ration, which lowers ruminant greenhouse gas production and promotes a more appropriate ecosystem while having no negative effect on fermentative properties. Further studies should test similar conditions in on-farm conditions.

## Conclusion

The treatment Mix. which includes a combination of the leaf extracts of the plant treatments AZI and CNA achieved the greater sustainable *in vitro* reduction, 67.6% in CH_4_ production when compared to either AZI or CNA alone. A concentration of 72 mg/g DM incubated of the mix was the best dosage for lowering *in vitro* ruminant GHG production while having no negative effect on total gas production. Thus, suggesting a potential synergistic effect among the phytochemical compounds from each plant species. It is important to note, however, that *in vitro* studies cannot always be directly extrapolated to *in vivo* conditions. Therefore, results should be confirmed with studies. In addition, future studies should consider evaluation of the ruminal microbial community and the complete volatile fatty acid profile.

## Data availability statement

The raw data supporting the conclusions of this article will be made available by the authors, without undue reservation.

## Author contributions

ME: conceptualization, methodology, formal analysis, investigation, resources, data curation, writing—review and editing, visualization, and project administration. NA-L and TA: conceptualization, methodology, writing—review and editing, supervision, project administration, and funding acquisition. UI: conceptualization, methodology, data curation, and writing—review and editing. RP: conceptualization, methodology, formal analysis, investigation, resources, data curation, writing—original draft preparation, writing—review and editing, visualization, supervision, project administration, and funding acquisition. EC-L: conceptualization writing—original draft preparation, and writing—review and editing. AS and MB-R: conceptualization, methodology, resources, data curation, writing—review and editing, supervision, project administration, and funding acquisition. All authors contributed to the article and approved the submitted version.

## Funding

The authors appreciate the financial support from Universidad Autónoma del Estado de México (Project UAEM 4304/2017/CI).

## Conflict of interest

The authors declare that the research was conducted in the absence of any commercial or financial relationships that could be construed as a potential conflict of interest.

## Publisher’s note

All claims expressed in this article are solely those of the authors and do not necessarily represent those of their affiliated organizations, or those of the publisher, the editors and the reviewers. Any product that may be evaluated in this article, or claim that may be made by its manufacturer, is not guaranteed or endorsed by the publisher.
